# Clinical and genetic analyses of premature mitochondrial encephalopathy with epilepsia partialis continua caused by novel biallelic *NARS2* mutations

**DOI:** 10.3389/fnins.2022.1076183

**Published:** 2022-12-21

**Authors:** Wenjing Hu, Hongjun Fang, Yu Peng, Li Li, Danni Guo, Jingwen Tang, Jurong Yi, Qingqing Liu, Wei Qin, Liwen Wu, Zeshu Ning

**Affiliations:** ^1^Department of Neurology, Hunan Children’s Hospital, Changsha, China; ^2^Pediatrics Research Institute of Hunan Province, Hunan Children’s Hospital, Changsha, China; ^3^Department of Radiology, Hunan Children’s Hospital, Changsha, China

**Keywords:** *NARS2*, COXPD24, aminoacylation reaction, mitochondrial disease, gene testing, minigene

## Abstract

Biallelic *NARS2* mutations can cause various neurodegenerative diseases, leading to growth retardation, intractable epilepsy, and hearing loss in early infancy and further progressing to spastic paraplegia, neurodegeneration, and even death. *NARS2* mutations are associated with mitochondrial dysfunction and cause combined oxidative phosphorylation deficiency 24 (COXPD24). Relatively few cases have been reported worldwide; therefore, the pathogenesis of COXPD24 is poorly understood. We studied two unrelated patients with COXPD24 with similar phenotypes who presented with intractable refractory epilepsia partialis continua, hearing loss, and growth retardation. One patient died from epilepsy. Three novel *NARS2* variants (case 1: c.185T > C and c.251 + 2T > G; case 2: c.185T > C and c.509T > G) were detected with whole-exome sequencing. c.251 + 2T > G is located at the donor splicing site in the non-coding sequence of the gene. The minigene experiment further verified that c.251 + 2T > G caused variable splicing abnormalities and produced truncated proteins. Molecular dynamics studies showed that c.185T > C and c.509T > G reduced the binding free energy of the *NARS2* protein dimer. The literature review revealed fewer than 30 *NARS2* variants. These findings improved our understanding of the disease phenotype and the variation spectrum and revealed the potential pathogenic mechanism of non-coding sequence mutations in COXPD24.

## 1 Introduction

Aminoacylation reaction refers to the formation of an ester bond between an amino acid and specific transfer RNA (tRNA), resulting in aminoacylated tRNA. It is involved in biological protein synthesis, mainly catalyzed by aminoacylated tRNA synthetase (aaRS) family enzymes (Wei et al., 2019). Based on enzyme catalytic domains and tRNA recognition sites, aaRS can be divided into classes I and II. Class II mainly performs biological functions in the form of homodimers (Banik and Nandi, 2012; Chaliotis et al., 2017). Asparaginyl tRNA synthetase (NARS) is a subtype of class II aaRSs, similar to most aaRSs. It includes the cytoplasmic type NARS1 and the mitochondrial type NARS2 (Vinogradova et al., 2021), both of which are associated with human neurodegenerative diseases. However, cytoplasmic and mitochondrial differences in protein localization and catalytic function can cause other diseases as well. Among them, heterozygous, biallelic *NARS1* mutations can lead to autosomal recessive neurodegenerative disorders, with microscopic, language, and gain abnormalities (OMIM#619091), and autosomal dominant neuropathological disorders, with microscopic, language, epilepsy, and gait abnormalities (OMIM#619092). The main clinical characteristics are growth retardation, seizures, peripheral neuropathy, and ataxia (Manole et al., 2020).

*NARS2* mutations can result in combined oxidative phosphorylation deficiency 24 (COXPD24, OMIM#616239). The main clinical characteristics are infantile seizures (which may progress to intractable epileptic retardation), overall growth retardation, mental retardation, hypotonia, and hearing impairment. Some cases may be accompanied by kidney disease or liver abnormalities (Simon et al., 2015; Sofou et al., 2015; Mizuguchi et al., 2017; Seaver et al., 2018; Štěrbová et al., 2021; Zhang et al., 2022). Compared to *NARS1* mutations, *NARS2* mutations are associated with earlier onset and worse condition of related nervous system diseases. To date, fewer than 30 *NARS2* variants have been reported worldwide; therefore, the disease and the variation spectrum should be further explored (Štěrbová et al., 2021).

Herein, we report two infants with premature mitochondrial encephalopathy with epilepsia partialis continua (EPC). Genetic testing revealed novel compound heterozygous *NARS2* mutations, including a rare intron mutation at the donor splicing site. The minigene experiment verified that the mutation led to abnormal splicing. The results of this study improved our understanding of the phenotype and the variation spectrum of COXPD24 and indicated that compound heterozygous *NARS2* mutations are involved in the pathogenesis of nervous system disorders.

## 2 Materials and methods

### 2.1 Patients

From 2019 to 2021, two patients belonging to two unrelated families in China were recruited from the Department of Neurology of Hunan Children’s Hospital. Their clinical manifestations, including disease history, physical examination, and brain magnetic resonance imaging (MRI) findings, were collected. The study protocol was approved by the Ethics Committee of Hunan Children’s Hospital.

### 2.2 Genetic testing

Trio-whole exome sequencing (trio-WES) was performed on the patients and their parents. We collected 3 mL of peripheral blood from each core family member. After anticoagulation treatment with ethylene diamine tetraacetic acid, we extracted leukocyte DNA according to the manufacturer instructions of the genome extraction kit, built the library, and captured the designed sequence with the Illumina NovaSeq 6000 high-throughput sequencer (Illumina, San Diego, CA, United States). We checked thousands of human genomes for the selected suspicious mutations in the normal population using the Single Nucleotide Polymorphism Database (dbSNP^1^), Exome Aggregation Consortium (ExAC^2^), and 1000 Genomes^3^. We predicted and analyzed the hazard of the variant spectrum using the online software Sorting Intolerant From Tolerant (SIFT^4^), Polyphen2^5^, and MutationTaster^6^. The pathogenicity of the variants was rated according to American College of Medical Genetics and Genomics (ACMG) guidelines (Richards et al., 2015). Finally, the primers were designed according to the Ensemble database^7^, and the variants were verified through Sanger sequencing using the Applied Biosystems 3500xL analyzer.

### 2.3 Conservative analysis

The human NARS2 protein (ID: Q96I59) and amino acid sequences of different species were retrieved from the Uniprot database^8^, and the conservatism analysis was performed using the Align function provided by the database.

### 2.4 Construction of a three-dimensional model of *NARS2* protein and molecular dynamics (MD) analysis

The NARS2 protein sequence was obtained from the National Center of Biotechnology Information^9^, and the Blast function^10^ was used for template retrieval. Modeller 9.15^11^ was used for single template modeling (PDB ID: 6PQH, Asparagine tRNA ligase of Elizabethkingia sp.). The sequence consistency was 40.77%, and the confidence level was 99.9%. The mutant structure was constructed on the wild-type structure using Pymol version 2.2. MD simulation was performed for wild-type and mutant variants with the GROMACS 5.1.4 package^12^ under constant pressure and using a heating system for 10 ns. Protein structure parameters were obtained using the Amber ff99SB force field. During simulation, the van der Waals force was calculated using a truncation threshold, with the non-truncation distance set as 1.4 nm. The particle mesh Ewald method was selected to calculate the electrostatic interaction. The linear constraint solver algorithm was used to constrain the chemical bonds formed by hydrogen atoms. The Verlet leapfrog algorithm was used to solve the Newton’s equation of motion at each step. The time step was 2 fs, and the Parrinello–Rahman method was used to control the pressure at 1 atm. After minimizing the energy with simulation, Pymol version 2.2 was used to display the results.

### 2.5 Minigene experiment for c.251 + 2T > G splicing function

A minigene plasmid was designed with three target fragments to be inserted into the genome sequence region of exons 1–3 of *NARS2*. Table 1 shows the primer information. The 5′-UTR region in exon 1 was removed; the 381-bp sequence in intron 1 was retained at the 5′ end; the 416-bp sequence in intron 1 was retained at the 3′ end and merged into a small region in intron 1; the 627-bp sequence in intron 2 was retained at the 5′ end; and the 366-bp sequence in intron 2 was retained at the 3′ end and merged into a small region in intron 2. The amplified product was cloned into the pMini CopGFP vector (Hitachi Biotechnology Co., Ltd., Beijing) digested by restriction endonuclease 5′ *Bam*HI/3′ *Xho*I using the ClonExpress II One Step Cloning Kit (Vazyme, Nanjing, China). Mutant plasmids were obtained by site-directed mutagenesis of wild-type plasmids through the primer design. The wild-type and mutant minigene plasmids verified by Sanger sequencing were transiently transfected into human embryonic kidney 293T cells with Lipofectamine 2000 (Invitrogen, Carlsbad, CA, United States). After 48 h, total RNA was extracted from cells using the TRIzol reagent (Cowin Biotech Co., Jiangsu, China). Subsequently, primers were designed for reverse transcription polymerase chain reaction amplification, followed by Sanger sequencing, and gene subtypes were determined.

**TABLE 1:** Primer information in the minigene experiment.

Wild-type plasmid	Primer sequence
NARS2-WT-AF	5′-AAGCTTGGTACCGAGCTCGGATCCAT GCTGGGGGTCCGCTGCCTGCTGCGGT-3′
NARS2-WT-AR	5′-TCTCACTGCGGTGTGAATACAAAGG CAGGTAAGACAC-3′
NARS2-WT-BF	5′-GTATTCACACCGCAGTGAGATGAA ACCAGAAAGAAG-3′
NARS2-WT-BR	5′-TTAAACGGGCCCTCTAGACTCGAGCTTGGC ATCACAATTTCCAATAACTTTAA-3′
NARS2-WT-CF	5′-TTATGGTCTCCAAAAATAGGACAG AGAATTCCCGGC-3′
NARS2-WT-CR	5′-TTAAACGGGCCCTCTAGACTCGAGC TTGGCATCACAATTTCCAATAACTTTAA-3′
Mutant plasmid	
NARS2-MUT-F	5′-ACAGTAGGgGAGTTTTGTTTTT TAAAAGAATTCTTTGA-3′
NARS2-MUT-R	5′-CAAAACTCcCCTACTGTCAA GGCCTGAATCTG-3′
RT-PCR	
MiniRT-F	5′-GGCTAACTAGAGAACCCACTGCTTA-3′
NARS2-RT-R	5′-CTTGGCATCACAATTTCCAATAACTTTAA-3′

### 2.6 Literature review

Using PubMed^13^ and Web of Science^14^ databases, retrospective statistical analyses of *NARS2* mutations and phenotypes were performed.

## 3 Results

### 3.1 Clinical features

#### 3.1.1 Patient #1

**FIGURE 1:**
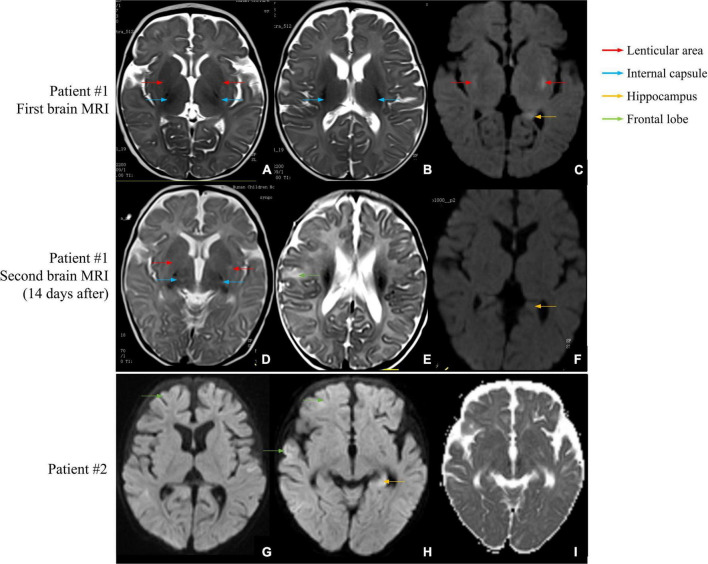
Brain MRI scans of patients #1 and #2. **(A–C)** First brain MRI examination of patient #1. **(A,B)** The T2-weighted image shows a small and slightly long T2 signal shadow in bilateral internal capsules and lenticular nuclei. **(C)** The diffusion-weighted image shows small patches of a slightly long T2 signal shadow in the upper outer capsule and left hippocampus. **(D–F)** Second brain MRI examination of patient #1. **(D)** The T2-weighted image shows a wider T2 signal shadow in bilateral internal and external capsules with higher signal intensity. **(E)** The T2 signal intensities are higher in the right frontal cortex and subcortical area than in the front. **(F)** No obvious abnormal signal is found in bilateral hippocampal regions, and no diffusion limitation is found in the lesions. **(G–I)** Brain MRI examination of patient #2. **(G)** The diffusion-weighted image shows wider and deeper sulcus fissures in bilateral cerebral hemispheres and slightly higher cortical signal in part of the cerebral hemispheres. **(H)** Partial cortex of the cerebral hemisphere and the left hippocampus show slightly high signal intensities. **(I)** The apparent diffusion coefficient map shows a slightly low signal intensity, indicating limited dispersion.

**FIGURE 2:**
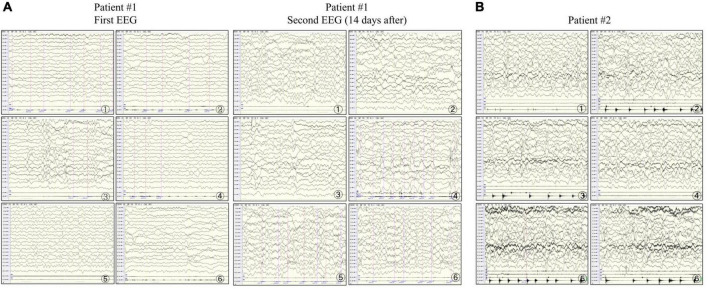
Electroencephalograms of patients #1 and #2. **(A)** In patient #1, the first examination shows abundant δ-wave activity in the left and midline regions of the brain. The sharp waves in each area (significant on the left) are synchronized or not synchronized with a few more sharp waves. The second examination shows a slowed background rhythm and more δ-wave activities in the right and midline regions of the brain. The frontal, central, parietal, occipital, and temporal areas (significant on the right) and the midline show numerous sharp slow waves, consistent with epilepsia partialis continua (EPC). **(B)** In patient #2, the right upper limb twitches rhythmically 1–2 times/s. The electroencephalogram synchronizes with the low to medium amplitude spike wave rhythm in the parietal, occipital, middle, and posterior temporal regions (significant on the right) in the background of diffuse rhythm, which can affect the central region, and the twitches are synchronized with spike waves, consistent with EPC.

Patient #1 was a 2-month-old girl with no adverse prenatal or perinatal history. She was admitted to the hospital because of seizures without inducement. Physical examination on admission showed clear consciousness, poor fixation and chasing abilities, no eye contact, insensitivity to external sound stimulation, hypotonia, and no head control. In the initial stage of seizures, her eyes titled repeatedly to the right, and the right side of the cheek and the right limbs twitched. Subsequently, the seizure progressed to general tremors. Two weeks later, during seizures, the head tilted to the left, and mainly the left limbs twitched. Auxiliary examination revealed no abnormalities. The auditory brainstem response (ABR) suggested that the bilateral auditory conduction pathways were involved, particularly in the central segment. Brain MRI showed abnormal signal shadows in the internal and external capsules and the left parahippocampal gyrus. Two weeks later, brain MRI showed a wider range of T2 signal foci in the internal and external capsules than in the front and similar signal changes in some areas of the right frontal cortex and under the cortex (Figures 1A–F). In the video electroencephalogram (VEEG), the background rhythm slowed down, and sharp waves were emitted in the central, top, occipital, and midline electrodes (Figure 2A). The parents were in good health, reported no family history of epilepsy, and had a non-consanguineous marriage. The antiepileptic treatment was performed with phenobarbital, topiramate, and levetiracetam. However, the patient still developed repeated convulsions, although with a low frequency. After discharge, levetiracetam combined with topiramate continued to be administered, but the treatment efficacy was poor, particularly when the frequency of attacks increased. Motor and mental developments showed degenerative changes. The patient died at the age of 11 months.

#### 3.1.2 Patient #2

Patient #2 was a 5-month-old girl with no adverse prenatal or perinatal history. The motor development milestone was delayed. At the age of 5 months, her head was still unstable, and she could not sit on her own. She was admitted to the hospital because of frequent convulsions. Physical examination on admission showed that she could not focus on or follow objects, made no eye contact, was insensitive to sound stimulation, and showed hypotonia. At the onset of the seizure, she showed twitching at the right corner of the mouth, blinking of the right eye, and response to call. The seizure lasted for approximately 2 h and was spontaneously relieved afterward. During hospitalization, she developed multiple epileptic seizures, manifesting as the body leaning back and the mouth corner tilting. Auxiliary examination showed blood lactic acid elevation (5.5 mmol/L; reference range: 0.5–1.7). ABR suggested that the bilateral auditory conduction pathways were involved. Brain MRI showed that the bilateral cerebral hemisphere sulcus fissures had widened and deepened. Further, the bilateral frontotemporal extra cerebral spaces had widened slightly, and the bilateral lateral ventricles had enlarged slightly. Diffusion-weighted imaging showed slightly high signal intensities at the partial cortex of the cerebral hemisphere and the left hippocampus (Figures 1G–I). VEEG during the epileptic attack suggested that in the background of a diffuse rhythm, the top, occipital, and middle and posterior temporal electrodes (mostly right side) showed a low-to-medium amplitude spike wave rhythm, affecting the central region (Figure 2B). The parents were in good health, reported no family history of epilepsy, and had a non-consanguineous marriage. The antiepileptic treatment was performed with levetiracetam and topiramate, combined with idebenone and levocarnitine to treat mitochondrial disease. However, the therapeutic efficacy was poor.

The phenotypes of the two patients were relatively similar and included intractable EPC occurring in early life, hypotonia, positive ABR, and comprehensive psychomotor retardation.

### 3.2 Trio-WES for *NARS2* variants

**FIGURE 3:**
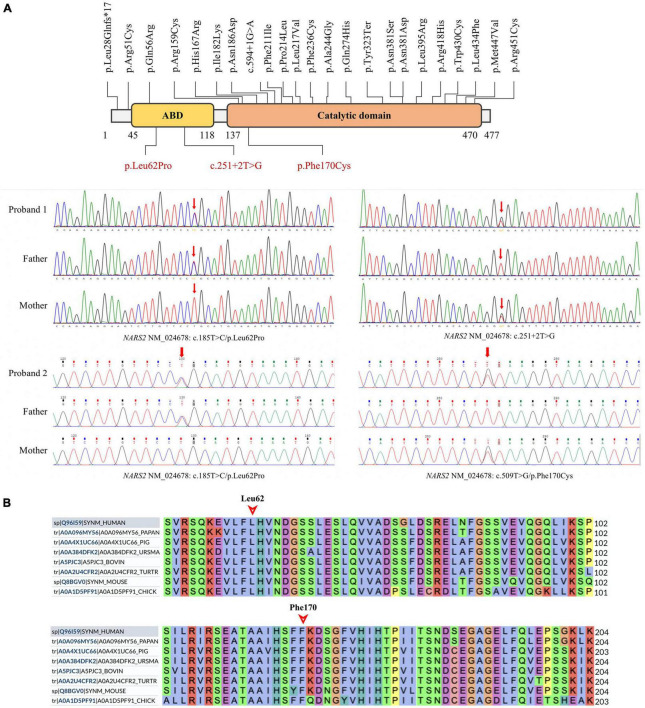
*NARS2* mutation information. **(A)** Including the three mutations in this study (red font), 26 *NARS2* mutations have been reported, mainly concentrated in the categorical domain, with the highest proportion of missense mutations. *NARS2* of patient #1 has compound heterozygous mutations NM_024678: c.185T > C/p.Leu62Pro and c.251 + 2T > G. The p.Leu62Pro is inherited from the father, and c.251 + 2T > G is inherited from the mother. Patient #2 also has compound heterozygous mutations NM_024678: c.185T > C/p.Leu62Pro and c.509T > G/p.Phe170Cys. The p.Leu62Pro is inherited from the father, and p.Phe170Cys is inherited from the mother. Sanger sequencing confirms the existence of mutations. **(B)** The homology analysis shows that Leu62 and Phe170 of the NARS2 protein are conservative among different species.

Patient #1 is compound heterozygous for c.185T > C/p.Leu62Pro and c.251 + 2T > G *NARS2* variations (NM_024678). p.Leu62Pro was inherited from the father, and c.251 + 2T > G was inherited from the mother. Patient #2 is compound heterozygous for c.185T > C/p.Leu62Pro and c.509T > G/p.Phe170Cys *NARS2* variations (NM_024678). p.Leu62Pro was inherited from the father, and p.Phe170Cys was inherited from the mother (Figure 3A). The dbSNP, ExAC, and 1000 Genomes showed a low frequency of p.Leu62Pro and no reports of c.251 + 2T > G or c.509T > G. SIFT. Polyphen2 and MutationTaster were harmful to the prediction results of mutation c.185T > C and c.509T > G (Table 2). According to ACMG guidelines, c.185T > C, c.251 + 2T > G, and c.509T > G are rated as “uncertain significance,” “likely pathetic,” and “uncertain significance,” respectively. Sanger sequencing confirmed the existence of each mutation. The homology analysis showed that the NARS2 proteins Leu62 and Phe170 were conservative among different species (Figure 3B).

**TABLE 2:** *NARS2* mutation information in this study.

Gene	*NARS2*	*NARS2*	*NARS2*
Transcript	NM_024678	NM_024678	NM_024678
Variant	c.185T > C/p.Leu62Pro	c.251 + 2T > G	c.509T > G/p.Phe170Cys
Chromosome	chr11:78282446-A-G	chr11-78282378-A-C	chr11:78277182-A-C
Provean	Deleterious (−5.82)	/	Deleterious (−7.91)
SIFT	Damaging (0.0)	/	Damaging (0.0)
Polyphen2_HDIV	Probably damaging (1.0)	/	Probably damaging (1.0)
Polyphen2_HVAR	Probably damaging (0.999)	/	Probably damaging (1.0)
MutationTaster	Disease_causing (1)	Disease_causing (1)	Disease_causing (1)
M-CAP	Damaging (0.094197)	/	Damaging (0.137594)
REVEL	Deleterious (0.798)	/	Deleterious (0.970)
dbSNP	rs1287544800	/	/
ExAC	0	0	0
1000 Genomes	0	0	0
ACMG rating	Uncertain significance	Likely pathogenic	Uncertain significance
Rating evidence	PM2_Supporting + PP3	PVS1 + PM2_Supporting	PM2_Supporting + PP3

### 3.3 Structural analysis of the mutant *NARS2* proteins Leu62Pro and Phe170Cys

**FIGURE 4:**
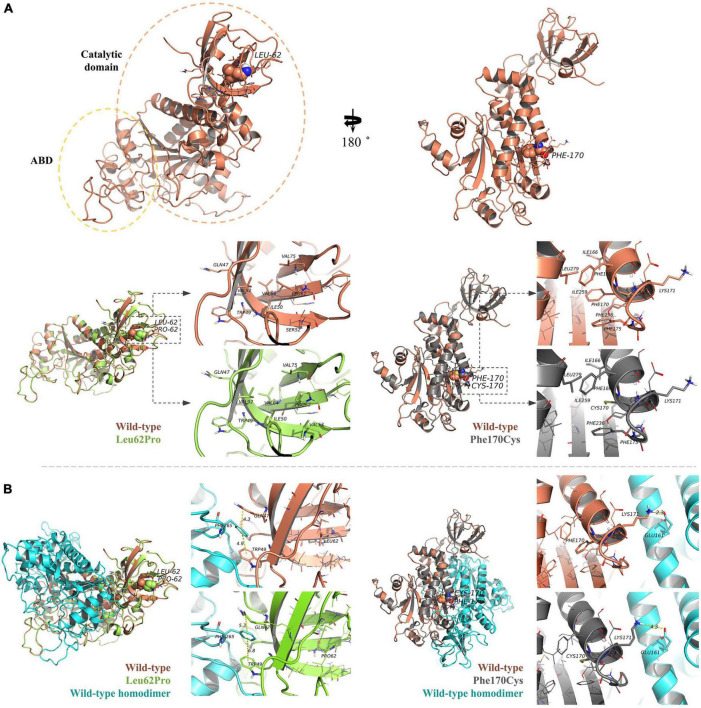
Protein structure prediction analysis. **(A)** The human NARS2 protein is composed of anticodon-binding and catalytic domains and functions as a homodimer. Wild-type Leu62 (in brown) is located in a pocket, surrounded by a hydrophobic group in the deep part of the protein, and forms a hydrophobic interaction with surrounding residues. Mutant Leu62Pro (green) may weaken the hydrophobic interaction with Val92. Another mutant Phe170Cys (black) is located in the pocket structure surrounded by hydrophobic groups, possibly weakening the interaction between Lys171 and Glu161 in the homodimer. **(B)** Molecular dynamics simulations predict that the mutation is affected the stability of the dimer structure. In the wild-type homodimers, Gln47 and Trp49 have formed stable interactions with the dimer Phe265, with distances of 4.3 and 4.9 angstroms (Å), respectively, while mutant Leu62Pro has weakened the interaction force, with distances of 5.3 and 6.8 Å, respectively. In the wild-type homodimer, Lys171 forms a hydrogen bond with the dimer Glu161, with a distance of 2.3 Å. In mutant Phe170Cys, the distance is 4.3 Å.

The human NARS2 protein is composed of 477 amino acids, which contains an-binding domain (ABD) and a catalytic domain. It exerts biological activity as a homodimer (Vinogradova et al., 2021). Wild-type Leu62 is located in a pocket, surrounded by a hydrophobic group in the deep part of the protein, and forms hydrophobic bonds with surrounding residues. Mutant Leu62Pro may weaken the hydrophobic interaction with Val92 and reduce the interaction of Gln47 with Trp49 and Phe265 in the homodimer. In this study, MD predicted that the binding free energy of mutant Leu62Pro and the homodimer were lower than those of the wild type (Table 3). Another mutant Phe170Cys was located in the pocket structure surrounded by hydrophobic groups, possibly weakening the interaction between Lys171 and Glu161 in the homodimer. MD showed that the binding free energy of mutant Phe170Cys and the homodimer were lower than those of the wild type (Figure 4).

**TABLE 3:** Binding energy of the *NARS2* protein dimer affected by mutations.

Energy (kJ/mol)	Wild-type	Leu62Pro	Phe170Cys
van der Waals energy	-175.4132	-162.999	-158.759
Electrostatic energy	-68.4732	-69.7393	-48.652
Polar solvation energy	172.9168	203.533	173.7712
Solvent-accessible surface area energy	-3.6468	-14.6844	-14.8136
Solvent-accessible voids energy	0	0	0
Weeks–Chandler–Andersen energy	0	0	0
Binding energy	-74.6164	-43.8903	-48.4537

### 3.4 Abnormal messenger RNA (mRNA) splicing caused by c.251 + 2T > G

**FIGURE 5:**
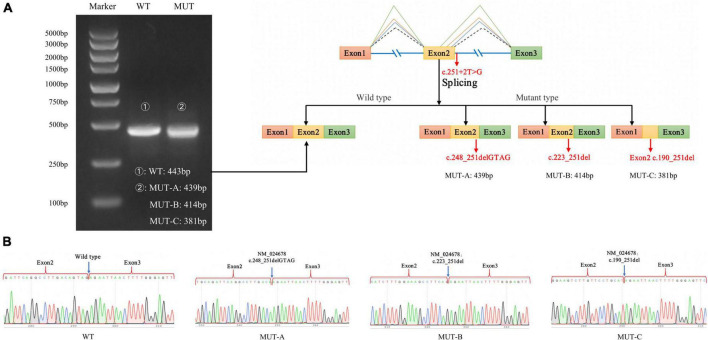
Minigene experiment results of the *NARS2* mutation c.251 + 2T > G. **(A)** Electrophoresis results of RT-PCR products of wild-type and mutant NARS2. The mutation plasmid c.251 + 2T > G produced three mRNA products, which were differentiated by cloning and sequencing because of the close distance between bands; **(B)** Sanger sequencing results of corresponding bands of wild-type and three mutant products.

As expected, the mRNA sequence of the wild-type plasmid included complete exons 1–3. The mutant plasmid produced three types of abnormal mRNA products through altered splicing. The first product was 4-bp deletion in exon 2 of mRNA, resulting in a new donor site (NM_024678: c.248_251delGTAG), and led to frameshift mutation (p.Ser83LysfsTer3). The second product was 29-bp deletion in exon 2 of mRNA, resulting in a new donor site (NM_024678: c.223_251del), and led to frameshift mutation (p.Val75ArgfsTer32). The third product was 62-bp deletion in exon 2 of mRNA, producing a new donor site (NM_024678: c.190_251del) and causing frameshifting mutation (p.Val64ArgfsTer32; Figure 5).

### 3.5 Literature review

We searched 14 articles related to neural performance affected by *NARS2* mutations (Simon et al., 2015; Sofou et al., 2015; Vanlander et al., 2015; Mizuguchi et al., 2017; Souza et al., 2017; Seaver et al., 2018; Han et al., 2019; Lee et al., 2020; Štěrbová et al., 2021; Tanaka et al., 2022; Vafaee-Shahi et al., 2022; Yagasaki et al., 2022; Yang et al., 2022; Zhang et al., 2022). A total of 21 patients and 23 *NARS2* mutations were reported in 16 families, most of which were missense mutations (91%, 21/23), followed by nonsense and frameshift mutations. Patients with biallelic *NARS2* mutations usually had bilateral sensorineural hearing impairments (75%, 15/20) in early life (first 3 months), followed by different types of seizures (95.6%, 22/23) within 1 year of age, mainly status epilepticus. Some cases rapidly progressed from local myoclonic to generalized seizures (Simon et al., 2015; Sofou et al., 2015). Approximately 88.8% (16/18) patients showed abnormal EEG performance, and approximately 77.2% (17/22) patients showed abnormal MRI findings, mainly diffuse brain atrophy. The antiepileptic treatment combining multiple antiepileptic drugs could not effectively control seizures in most patients. Some patients developed pentobarbital-induced burst suppression (Seaver et al., 2018) or Fanconi syndrome induced by valproic acid sodium (Tanaka et al., 2022). In addition, almost all patients showed developmental abnormalities to varying degrees, including growth retardation, severe intellectual disability, and mental and motor retardation. Some cases progressed to ataxia in a progressive and chronic manner. The mortality of patients with biallelic *NARS2* mutations was higher (26.1%, 6/23). Other manifestations, such as elevated blood or cerebrospinal fluid lactic acid and blood glucose, were occasionally reported (Table 4).

**TABLE 4:** Reported cases of nervous system abnormalities caused by *NARS2* mutations.

Case	Onset age	Sex	Nucleotide variant	Amino acid variant	Seizure type	Develop- ment	Neuro- logical/muscular disease	EEG	MRI	ABR	Auxiliary exami- nation	AED	Seizure control	Outcome	Ref.
1	8m	M	c.707T > G/c.594 + 1G > A	p.Phe236Cys/−	SE	GR, severe ID	Hypotonia	Diffuse spikes and slow-wave complexes	DCA	+	Elevated blood lactate	NA	NA	NA	Richards et al., 2015
2	10 m	F	c.707T > G/c.594 + 1G > A	p.Phe236Cys/−	ME	Developmental regression and ID	Hypotonia	Multifocal spikes	−	+	Elevated blood lactate	NA	NA	NA	
3	4 m	F	c.151C > T/c.1184T > G	p.Arg51Cys/p.Leu395Arg	SE	Developmental milestone delay	Hypotonia	Spikes and wave complexes in the left occipital area	DCA	+	Elevated blood lactate	NA	NA	NA	
4	5 m	M	c.500A > G/c.500A > G	p.His167Arg/p.His167Arg	ME + SE	Developmental milestone delay	Hypotonia	Burst suppression pattern	Cerebral atrophy with extended vacuolization of the periventricular WM, BG, CC, and cerebellum	+	Elevated blood lactate	NA	NA	NA	
5	3 m	M	c.167A > G/c.631T > A	p.Gln56Arg/p.Phe211Ile	SE	NA	Hypotonia	Epileptiform discharges over the left centro- temporal leads	Abnormal T2 hyperintensity at the posteromedial and parietal OLs and cerebral WM DCA	−	Elevated platelet count	PB + KD	PB-induced burst suppression	Death from epilepsy with multiple organ failures at the age of 5 months	Simon et al., 2015
6	4 m	M	c.167A > G/c.631T > A	p.Gln56Arg/p.Phe211Ile	SE		Hypotonia	Electrographic seizures from the left centroparietal region	Progressive WT T2 hyperintensity, bifrontal subdural effusions, and widespread cerebral restricted diffusion	−		PB + KD	PB-induced burst suppression	NA	
7	1 m	M	c.969T > A/c.1142A > G	p.Tyr323Ter/p.Asn381Ser	Local ME, and rapidly developed into generalized ME	NA	Hypotonia	+	Hyperintense T2-weighted and FLAIR signals within the periventricular WM and PCR with extension into the posterior limbs of the internal capsule	+	Elevated CSF lactate	TPM, PHT, CZP	Uncontrol- lable	Death from respiratory failure at the age of 15 months and autopsy findings of the brain structure consistent with Leigh syndrome	Mizuguchi et al., 2017
8	1 m	M	c.969T > A/c.1142A > G	p.Tyr323Ter/p.Asn381Ser	Local ME	NA	Hypotonia	+	Restricted diffusion in the left BG and external capsule junction and the left FL in cortical distribution	NA			NA	Death from respiratory failure at the age of 6 months and autopsy findings of the brain structure consistent with Leigh syndrome	
9	1 d	M	c.641C > T/c.641C > T	p.Pro214Leu/p.Pro214Leu	ME,TCS,AE	Global developmental delay, motor milestone delay, severe ID	Hypotonia	Bilateral synchronous spikes and polyspike waves	Profound supratentorial atrophy of the cerebral cortex, complete agenesis of the CC, and hypomyeli- nation of the WM	NA	Metabolic alkalosis and elevated CSF and blood lactate	VPA, BZO		Death at the age of 16	Seaver et al., 2018
10			c.731C > G/c.1351C > T	p.Ala244Gly/p.Arg451Cys	NA	NA	NA	NA	NA	NA	NA	NA	NA	NA	Tanaka et al., 2022
11	21 y	M	c.822G > C/c.822G > C	p.Gln274His/p.Gln274His	+	Dysarthria, ID	Amyotrophy and Babinski sign	NA	NA	+	NA	NA	NA	Slow progression to ataxia	Vafaee-Shahi et al., 2022
12	NA	NA	c.1253G > A/c.1300C > T	p.Arg418His/p.Leu434Phe	NA	NA	NA	NA	NA	NA	NA	NA	NA	NA	Vanlander et al., 2015
13	34 y	F	c.822G > C/c.822G > C	p.Gln274His/p.Gln274His	−	Language development delay	Proximal muscle weakness, severe amyotrophy, facial muscle paralysis	−	−	−	−	−	−	−	Vinogradova et al., 2021
14	NA	M	c.822G > C/c.822G > C	p.Gln274His/p.Gln274His	+	ID	−	−	−	−	−	−	−	−	
15	3.5 m	M	c.83_83del/c.1339A > G	p.Leu28Glnfs *17/p.Met447Val	ME and paroxysmal epilepsy with upward strabismus	Motor milestone delay	Hypotonia	Multifocal spikes and sharp waves, continuous focal sharp waves over the right or left frontal or temporo-occipital regions	Atrophy of the cortex and the periventricular brain	NA	−	PB, MZ, LEV	Poor control	Death from renal failure at the age of 14 months	Souza et al., 2017
16	6 m	M	c.1141A > G/c.1290G > C	p.Asn381Asp/p.Trp430Cys	GTC	Motor milestone delay	Hypotonia	Arrhythmic slow waves mixed with irregular spikes and sharp slow waves in the central, parietal, and temporal regions	−	+	Abnormal liver enzymes	VPA, PB, TPM, CZP	Decreased frequency	Death from epilepsy at the age of 6 months	Sofou et al., 2015
17	12 m	F	c.545T > A/c.545T > A	p.Ile182Lys/p.Ile182Lys	GTCS	Language development delay, slow progression to ataxia	NA	Bilateral synchronous spike and polyspike waves	−	+		PHB, NZP, LEV	Controllable	−	Wei et al., 2019
18	17 m	F	c.731C > G/c.556A > G	p.Ala244Gly/p.Asn186Asp	GTCS	Severe motor milestone and psychomotor development delay	Hypotonia	NA	DCA	+	VPA-induced Fanconi syndrome and metabolic abnorma- lities	VPA, PB, TPM, CZP, MZ	Poor control	Severe retardation of psychomotor development, without head control or visual tracking ability, and dysphagia; gastrostomy required	Yagasaki et al., 2022
19	8 m		c.1253G > A/c.1300C > T	p.Arg418His/p.Leu434Phe	ME	GR	Deep tendon reflexes and Babinski sign	NA	Bilateral, symmetric hyperintense lesions on T2-weighted and FLAIR MRI in the bilateral BG and lenticular nuclei	−	−	NA	NA	NA	Yang et al., 2022
20	6 m		c.475C > T/c.649T > G	p.Arg159Cys/p.Leu217Val	ME + TCS	NA	NA	Multifocal epileptiform activity and slowing of background activity	Hyperintense T2 signal at the BG	+	Hyperg- lycemia, reduced glycosylated hemoglobin, and elevated CSF and blood lactate	PHB, ethyl loflazepate	Poor control	Spastic paralysis, wheelchair required	Zhang et al., 2022
21	3 m		c.475C > T/c.649T > G	p.Arg159Cys/p.Leu217Val	Convulsion	Global developmental delay	NA	NA	DCA	+	Hyperg- lycemia, reduced glycosylated hemoglobin, and elevated CSF and blood lactate	PHB, ethyl loflazepate	Poor control	Wheelchair and gastrostomy required	

y, year; m, month; F, female; M, male; SE, status epilepticus; ME, myoclonic epilepsy; TCS, tonic clinic seizure; AE, absence epilepsy; GTCS, generalized tonic clinic seizure; GR, growth retardation; ID, intellectual disability; DCA, diffuse cerebral atrophy; CC, corpus callosum; BG, basal ganglia; WM, white matter; OL, occipital love; FL, frontal lobe; PCR, posterior corona radiata; FLAIR, fluid-attenuated inversion recovery; CSF, cerebrospinal fluid; AEDs, antiepileptic drugs; VPA, valproic acid; PB, pentobarbital; KD, ketogenic diet; TPM, topiramate; PHT, phenytoin; CZP, clonazepam; BZO, benzodiazepine; MZ, midazolam; LEV, levetiracetam; CZP, clonazepam; PHB, phenobarbital; NZP, nitrazepam; ABR, auditory brain stem; NA, not applicable.

## 4 Discussion

We reported two patients from two unrelated families with clinical characteristics of early-onset mitochondrial encephalopathy accompanied by EPC. The phenotypes of the infants were similar and included intractable EPC, hypotonia of the limbs, and general psychomotor retardation in early life. Brain MRI showed abnormal signals in the cortex and the hippocampus. Seizures in patient #1 was characterized by right limb twitching in the initial stage and left limb twitching 2 weeks later, not consistent with any epileptic type in previously reported cases. Three novel *NARS2* mutations (p.Leu62Pro, p.Phe170Cys, and c.251 + 2T > G) were identified with genetic testing, including one non-coding sequence mutation. Our structural analysis of the two missense mutations showed that the decreased local structural hydrophobicity affected the structural stability. MD results suggested the consequent decrease in the dimer binding free energy of the NARS2 protein, possibly affecting the protein function. The minigene experiment showed that c.251 + 2T > G affected mRNA splicing and causing frameshift mutations, which may lead to reduced protein expression or degradation. Taken together, clinical examination and genetic analysis of two patients revealed a diagnosis of COXPD24, the role of *NARS2* non-coding sequence mutations in the disease pathogenesis, and novel phenotype and mutation spectrum of COXPD24.

The disease phenotype spectrum resulting from biallelic *NARS2* mutations is relatively broad. Most infants diagnosed with COXPD24 have sensorineural hearing loss in the early stage, followed by delayed neural development, intractable seizures, and hypotonia. MRI shows white matter lesions, mainly involving the frontal and parietal lobes and the deep white matter, and features of agenesis of the corpus callosum or diffuse brain atrophy (Simon et al., 2015; Sofou et al., 2015; Vanlander et al., 2015; Mizuguchi et al., 2017; Souza et al., 2017; Seaver et al., 2018; Han et al., 2019; Lee et al., 2020; Štěrbová et al., 2021; Tanaka et al., 2022; Vafaee-Shahi et al., 2022; Yagasaki et al., 2022; Yang et al., 2022; Zhang et al., 2022). The characteristics of Leigh syndrome can be observed in some patients’ brain MRI or autopsy results, including symmetric long T2 abnormal signals in the brain stem or the putamen or atrophy of the corpus callosum with lamellar necrosis and vacuolization (Simon et al., 2015; Tanaka et al., 2022). In addition, a few cases may be accompanied by renal dysfunction or elevated liver enzymes, possibly leading to a clinical misdiagnosis of Alpers syndrome (Seaver et al., 2018). The survival rate of patients with COXPD24 is low. The reported cases have shown a mortality rate of approximately 26%. The survivors may gradually lose their ability to take care of themselves and usually develop chronic progressive ataxia, severe psychomotor retardation or regression, loss of head control, and dysphagia (Souza et al., 2017; Tanaka et al., 2022). More rare phenotypes also include neonatal diabetes mellitus and metabolic acidosis (Yagasaki et al., 2022). Therefore, customized nursing for survivors of COXPD24 may help improve the quality of life. However, the complex and variable phenotypic disease characteristics pose some challenges to the accurate clinical diagnosis. As for the genetic etiology, including the three novel mutations identified in this study, 26 *NARS2* mutations have been reported. However, according to the existing genotype–phenotype association analysis, whether the phenotype is non-progressive and mild or severe, infantile, and lethal cannot be determined. Further, no significant correlation seems to exist with the location or type of mutation (Sofou et al., 2015). However, this may also be because of the lack of sufficient genotype and phenotype data.

The *NARS2* locus on chromosome 11q14.1 spans 14 exon regions and encodes a protein structure consisting of ABD (amino acids 38–122) and catalytical domain (amino acid 137–471), lacking the unique N-terminal extension structure of the NARS1 protein (Zhang et al., 2022). The literature review revealed 23 *NARS2* mutations, most of which were missense mutations and mainly distributed in the catalytic domain region. In this study, three novel *NARS2* mutations were identified, including the two missense mutations Leu62Pro and Phe170Cys located in the ABD region and the catalytic domain, respectively. The crystal structure of the human NARS2 protein has not been analyzed. Molecular modeling data showed that wild-type Leu62 and Phe170 were located in hydrophobic pockets and adjacent to the homodimer structure. The mutations decreased the local structural hydrophobicity. MD predicted that the mutations Leu62Pro and Phe170Cys decreased the dimer binding free energy of the NARS2 protein, possibly indicating that the mutation affected protein dimerization. The results of the hazard prediction algorithm for amino acid changes caused by these point mutations also indicated the existence of deleterious and damaging.

*NARS2* non-coding sequence mutations have rarely been reported. Only one classical splice-site mutation has been reported in a pair of siblings with severe intellectual disability and status epilepticus (Mizuguchi et al., 2017). In this study, one patient with c.251 + 2T > G showed EPC at the age of 2 months, followed by progression to generalized seizures and death at the age of 11 months. This suggested that non-coding sequence mutations leading to mRNA splicing changes may aggravate the patient’s phenotype. c.251 + 2T > G is not included in the distribution frequency database of the normal population. The minigene experiment indicated that the mutation produced three mRNA products. Because of the small sequence length difference between these products, we differentiated them with cloning sequencing. The results showed that the three mRNA products led to truncated proteins. Thus, we strengthened the PS3 evidence of c.251 + 2T > G through experiments and further revealed that the non-coding sequence mutation played a role in the pathogenesis of COXPD24.

## 5 Conclusion

We performed genetic analyses of two infants with COXPD24 and identified three novel *NARS2* mutations. Both patients showed early-onset mitochondrial encephalopathy with EPC, bilateral sensorineural deafness, hypotonia, and growth retardation. With the wide application of genetic testing in neurogenetic diseases, the novel clinical phenotype of COXPD24 would be increasingly reported, suggesting that clinicians should pay more attention to the characteristics of aaRS. The non-coding sequence mutation found in this study was confirmed to be a biohazard in the minigene experiment. The structural model analysis suggested that two point mutations might affect protein dimerization. However, this study had some limitations. First, we could not explore the mitochondrial respiratory chain function in the patients’ tissue samples, thereby leading to the lack of a correlation analysis between the mutation and cell function. Second, we could not perform subcellular localization experiments for the novel mutations or verify the interference of the mutation with protein dimerization. Nevertheless, the findings of this study improve our understanding of the *NARS2* mutation profile and phenotype.

## Data availability statement

The datasets presented in this study can be found in online repositories. The names of the repository/repositories and accession number(s) can be found in the article/Supplementary material.

## Ethics statement

The studies involving human participants were reviewed and approved by the Ethics Committee of Hunan Children’s Hospital. Written informed consent to participate in this study was provided by the participants’ legal guardian/next of kin. Written informed consent was obtained from the individual(s), and minor(s)’ legal guardian/next of kin, for the publication of any potentially identifiable images or data included in this article.

## Author contributions

WH and HF carried out the data curation and formal analysis, and wrote the original draft of the manuscript. YP, LL, QL, and WQ carried out the data curation, visualized the data, did the conceptualization, and performed the methodology. DG, JT, and JY carried out the software and experimental validation. LW and ZN supervised the data and reviewed and edited the manuscript. All authors contributed to the article and approved the submitted version.

## Footnotes

1
www.ncbi.nlm.nih.gov/snp

2
https://gnomad.broadinstitute.org/

3
www.1000genomes.org

4
https://sift.bii.a-star.edu.sg/

5
http://genetics.bwh.harvard.edu/pph2/

6
http://www.mutationtaster.org

7
http://asia.ensembl.org/index.html

8
www.uniprot.org/

9
www.ncbi.nlm.nih.gov/nuccore/NM_024678.6/

10
https://blast.ncbi.nlm.nih.gov/Blast.cgi

11
www.salilab.org/modeller

12
www.gromacs.org/

13
www.pubmed.ncbi.nlm.nih.gov/

14
videos.webofsciencegroup.com/
