# Supplementary material for: Clinical and genetic analyses of premature mitochondrial encephalopathy with epilepsia partialis continua caused by novel biallelic NARS2 mutations
Source: Front Neurosci. 2022 Dec 21;16:1076183. doi: 10.3389/fnins.2022.1076183 (PMC9811187; doi:10.3389/fnins.2022.1076183)

Supplementary Material

1 Minigene experimental methods

1. The NARS2 (NM_024678: c.251+2T>G) wild type minigene plasmid and the NARS2 (NM_024678: c.251+2T>G) mutant minigene plasmid were constructed respectively. The construction information is as follows:

Clone vector: pMini CopGFP

Clone site: BamHI/XhoI

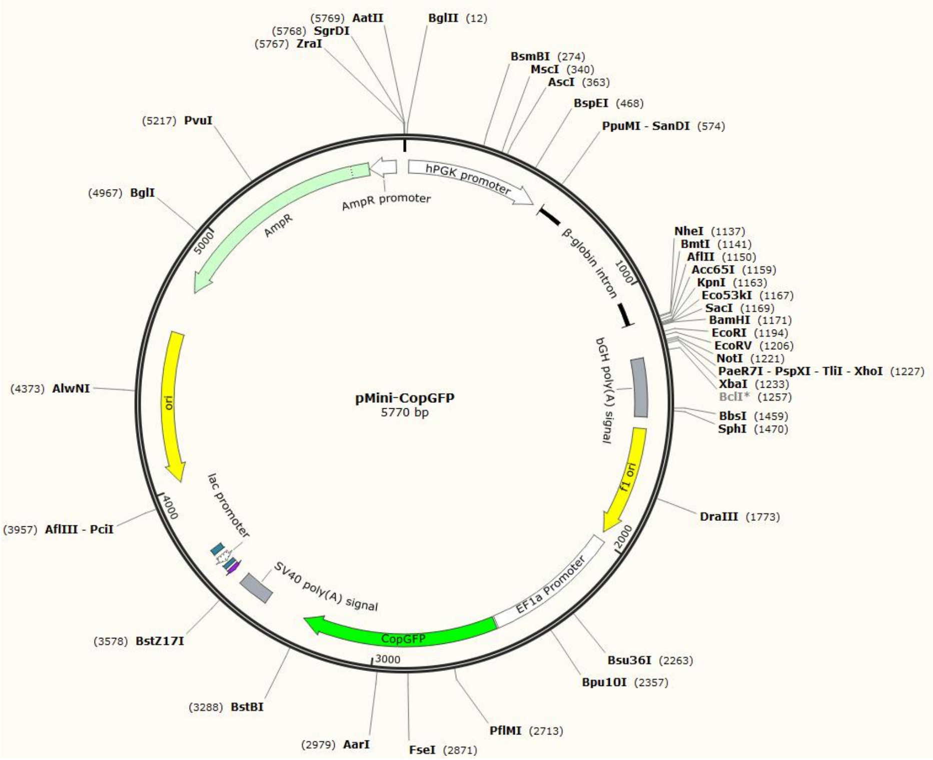

Insert sequence information:

NARS2-WT(NM_024678： c.251+2T>G)

atgctgggggtccgctgcctgctgcggtccgtgcgcttctgttcctccgccccccttccccaagcacaaaccttcagccaaactgagcgtgcggga
cgctctcggggctcagaacgcgagtggggagcgcattaagatccaggttggtgaatgggaagggagtgggtttttttcttgtagggacttttatatgg
cacatccacatctagcttttcttttacagtcgggtcagacacaaccgccagcagggaaaagttagaaagtgtaggcgtcgcgggtttgcacttttctg
atttccattacttttcacaaaactgttccgataactgtaaagcgctggaactttgctttcatgattccagggtattacctgtaaacttgttaatcataccaaa
agtgtaaatgaacaagccccctttctctatctttatcccttataatgaatatccaggtttataaagtggttgtcttgacctccctccttgagagcagaaacc
gtgtcttacctgcctttgtattcacaccgcagtgagatgaaaccagaaagaagggcgcaccccacatcctgagaaaggcaccaagatatgtgccct
gttatttattgttgggccagagggcagggctctgggaggaaatacctggaattgccaatattgggaggattgattatatgaattaggctcatcaacat
gaaggaatgttaggcatccattcgaaatgttgtgcattattacgaaaacaaaagatagaataccaaagtgtgtatcctattgtgattggaacatataaa
attaagtatgcatattggtgaactaaaagaagttgatttgttgaggcaggattgtgagtacatgttttactttcatttagtctgtgtgtgtatgtgtaatgaa

aacgtaaaatgttttacgctcacattggaaatatttctcttccttagggatggattcgttctgtccgatcccagaaggaagtcttgttcctgcatgtaaatg
atgggtcatctttggaaagccttcaggttgttgcagattcaggccttgacagtagggtgagttttgtttttaaaagaattctttgatttttgtttttcacctcta
gtgcttctcacttccctactccagtgtttacgttggacccctgagtaatcttttgaaacagatctgctttaatagctttgaggatcaactccgaatatttgg
aatcaagatccttcaagctcaggcctttcctcctctatttgacattgtcacatgctgatatcccctacttatgcttcacttcagtctttcttaattacctgcgg
ctcccttaagctgttctgctctttcctaatagcatttgtacatgttgctgcctctgcccaatacagcctcttctcctcttgtctttcatagttcctgcttagtta
ggcatcatctgatgttcccatcccatgattatgtcaggtacccctttttgtgctcccatagtgtcccgtgcctacatctgtcttaaaatgtatcacattgaa
tggtaatcaactgtattcctatttgaatcctcctgctagactcttaatttcctgaggttaggaatggcatctcactctttttgtattcttggcctctagcatag
tgctgacaatatgacaaattattgcctgttgaataaatggaagaaactttatggtctccaaaaataggacagagaattcccggcaaattttattttgattt
gcagttttcccatgtattctgtaatcttttaaaaagtgttttggccaactaactttttaataagggcaattgtacaacctcttaattctgaattaattatacaaa
ttcttaactctattcccattttaaagcttatggaattcattttataactttatgctctatttatacgacagattatccatagattctgaagattatttaacccaata
agggtaaaagcttttgtcattaagtattttaatattcttattgcaaagttgatattaaaatgaaggttgtatataatgatatcttgtttatcttgtttttgttactat
agagaattaaattttgggagttctgtggaagtacaagggcagctgataaaaagtccatccaaaaggcaaaatgtggaactgaaggcagaaaaaatt
aaagttattggaaattgtgatgccaag

NARS2-MT(NM_024678： c.251+2T>G)

atgctgggggtccgctgcctgctgcggtccgtgcgcttctgttcctccgccccccttccccaagcacaaaccttcagccaaactgagcgtgcggga
cgctctcggggctcagaacgcgagtggggagcgcattaagatccaggttggtgaatgggaagggagtgggtttttcttgtagggacttttatatgg
cacatccacatctagcttttcttttacagtcgggtcagacacaaccgccagcagggaaaagttagaaagtgtaggcgtcgcgggtttgcacttttctg
atttccattacttttcacaaaactgttccgataactgtaaagcgctggaactttgctttcatgattccagggtattacctgtaaacttgttaatcataccaaa
agtgtaaatgaacaagcccccttttctctatctttatcccttataatgaatatccaggtttataaagtggttgtcttgacctccctccttgagagcagaaacc
gtgtcttacctgcctttgtattcacaccgcagtgagatgaaaccagaaagaagggcgcaccccacatcctgagaaaggcaccaagatatgtgccct
gttatttattgttgggccagagggcagggctctgggaggaaatacctggaattgccaatattgggaggattgattatatgaattaggctcatcaacat
gaaggaatgttaggcatccattcgaaatgttgtgcattattacgaaaacaaaagatagaataccaaagtgtgtatcctattgtgattggaacatataaa
attaagtatgcatattggtgaactaaaagaagttgatttgttgaggcaggattgtgagtacatgttttactttcatttagtctgtgtgtgtatgtgtaatgaa
aacgtaaaatgttttacgctcacattggaaatatttctcttccttagggatggattcgttctgtccgatcccagaaggaagtcttgttcctgcatgtaaatg
atgggtcatctttggaaagccttcaggttgttgcagattcaggccttgacagtaggGgagttttgtttttaaaagaattctttgatttttgtttttcacctct
agtgcttctcacttccctactccagtgtttacgttggacccctgagtaatcttttgaaacagatctgctttaatagctttgaggatcaactccgaatatttg
gaatcaagatccttcaagctcaggcctttcctcctctatttgacattgtcacatgctgatatcccctacttatgcttcacttcagtctttcttaattacctgcg
gctcccttaagctgttctgctctttcctaatagcatttgtacatgttgctgcctctgcccaatacagcctcttctcctcttgtctttcatagttcctgcttagtt
aggcatcatctgatgttcccatcccatgattatgtcaggtacccctttttgtgctcccatagtgtcccgtgcctacatctgtcttaaaatgtatcacattga
atggtaatcaactgtattcctatttgaatcctcctgctagactcttaatttcctgaggttaggaatggcatctcactctttttgtattcttggcctctagcata
gtgctgacaatatgacaaattattgcctgttgaataaatggaagaaactttatggtctccaaaaataggacagagaattcccggcaaattttattttgatt
tgcagttttcccatgtattctgtaatcttttaaaaagtgttttggccaactaactttttaataagggcaattgtacaacctcttaattctgaattaattatacaa
attcttaactctattcccattttaaagcttatggaattcattttataactttatgctctatttatacgacagattatccatagattctgaagattatttaacccaat
aagggtaaaagcttttgtcattaagtattttaatattcttattgcaaagttgatattaaaatgaaggttgtatataatgatatcttgtttatcttgtttttgttact
atagagaattaaattttgggagttctgtggaagtacaagggcagctgataaaaagtccatccaaaaggcaaaatgtggaactgaaggcagaaaaa
attaaagttattggaaattgtgatgccaag

2. The flow chart of plasmid construction is as follows:

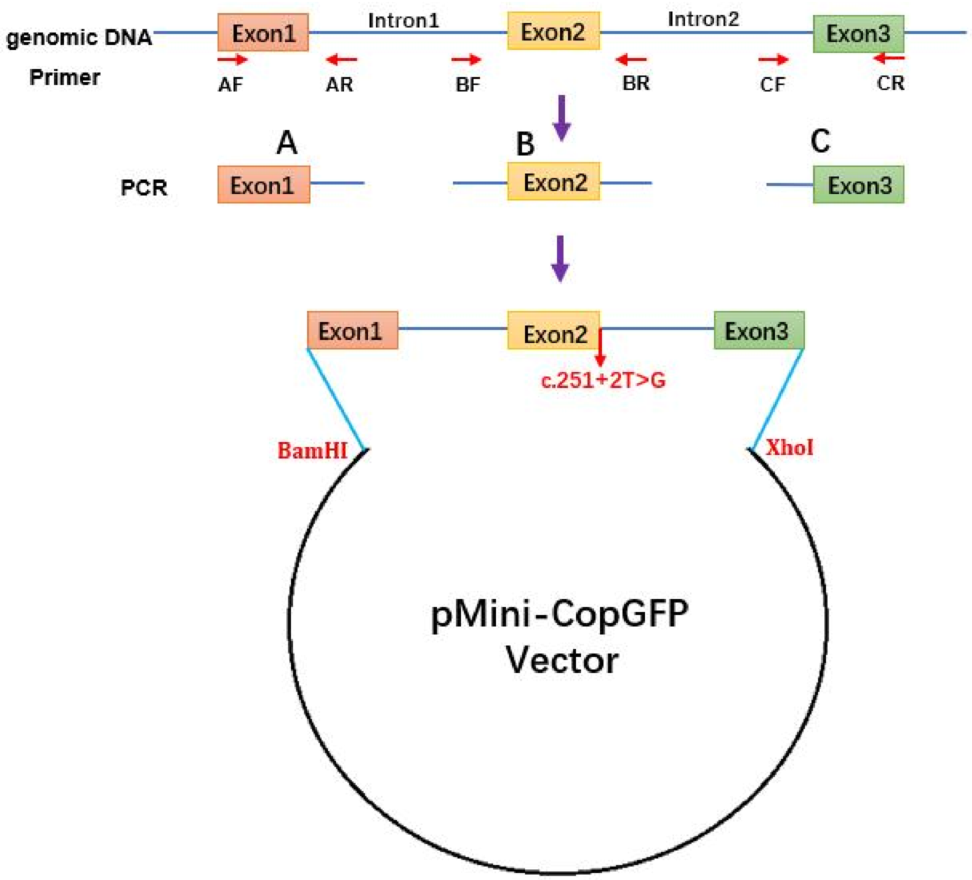

3. Primers were designed for RT-PCR amplification and product sequencing. The primer sequence is as follows:

MiniRT-F: GGCTAACTAGAGAACCCACTGCTTA

NARS2-RT-R: CTTGGCATCACAATTTCCAATAACTTTAA

2 Results

1. The electrophoretic chart is as follows:

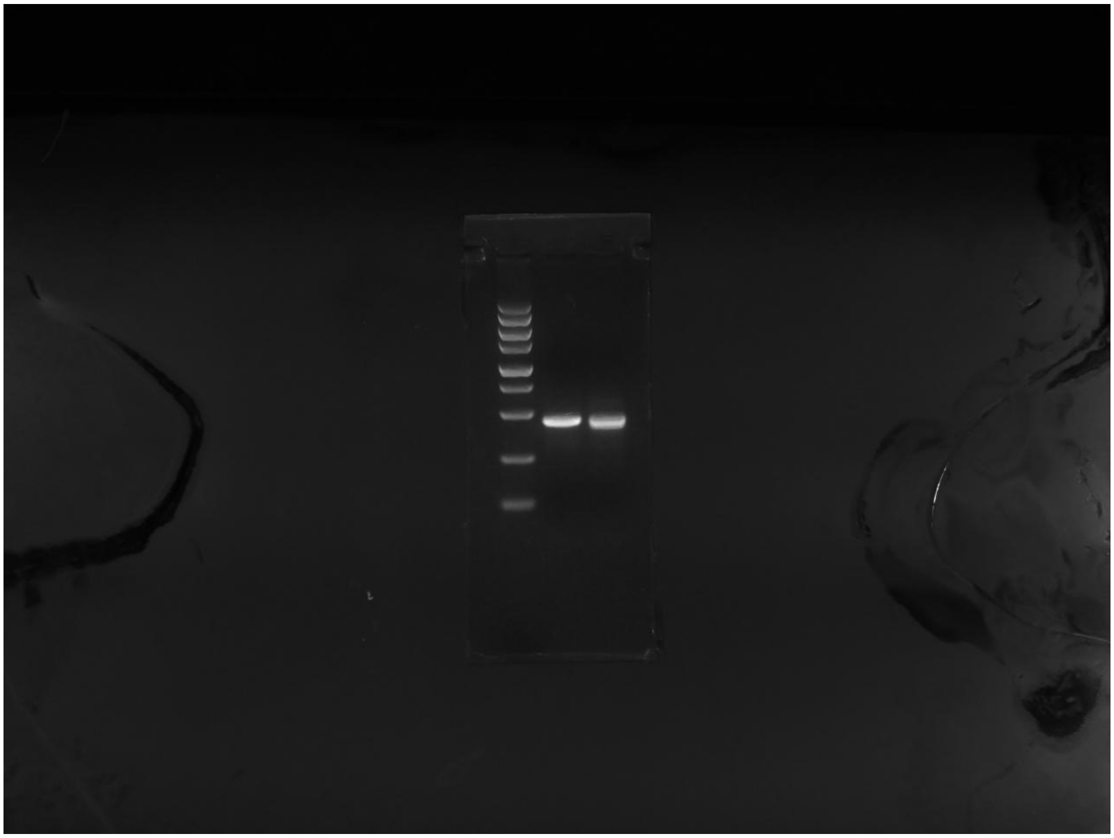

2.The RT-PCR product agarose gel was recycled and sent to Beijing Qingke Sanger for sequencing. The sequencing results are as follows:

WT：

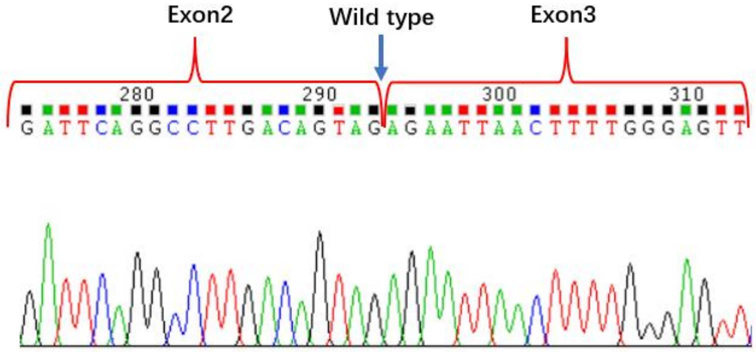

MT-A：

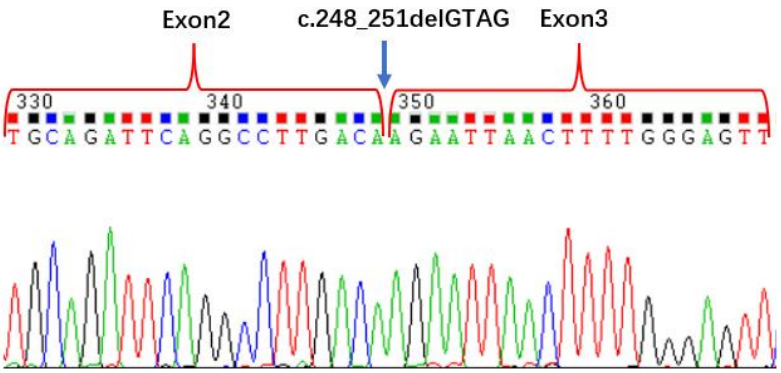

MT-B:

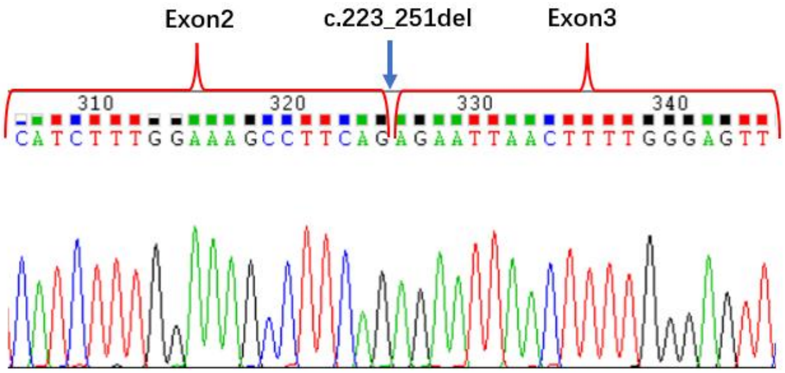

MT-C:

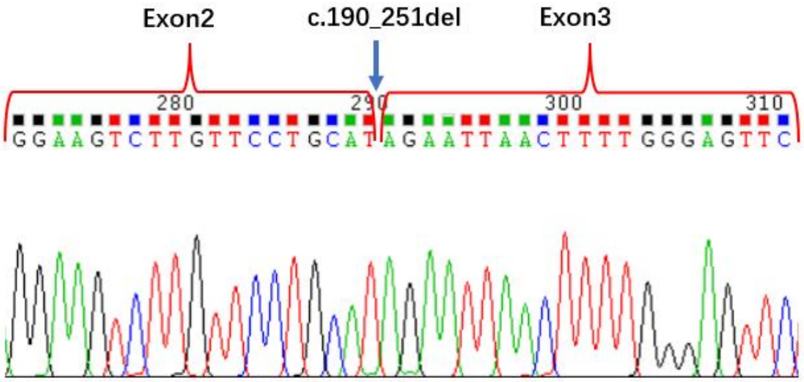

3. Result analysis

The expected sequence of normal group amplified by PCR is 443 bp: (the black font is the carrier transcriptional sequence, and the red font is the target gene exon transcriptional sequence):

GGCTAACTAGAGAACCCACTGCTTACTGGCTGCTAGCGTTTAAACTTAAGCTTGGTACCG
AGCTCGGATCCatgctgggggtccgctgcctgctgcggtccgtgcgcttctgttcctccgccccccttccccaagcacaaaccttcagcca
aactgagcgtgcgggacgctctcggggctcagaacgcgagtggggagcgcattaagatccagggatggattcgttctgtccgatcccagaagg

aagtcttgttcctgcatgtaaatgatgggtcatctttggaaagccttcaggttgttgcagattcaggccttgacagtagagaattaaattttgggagttct
gtggaagtacaagggcagctgataaaaagtccatccaaaaggcaaaatgtggaactgaaggcagaaaaaattaaagttattggaaattgtgatgc
caag

Electrophoretic chart identification:

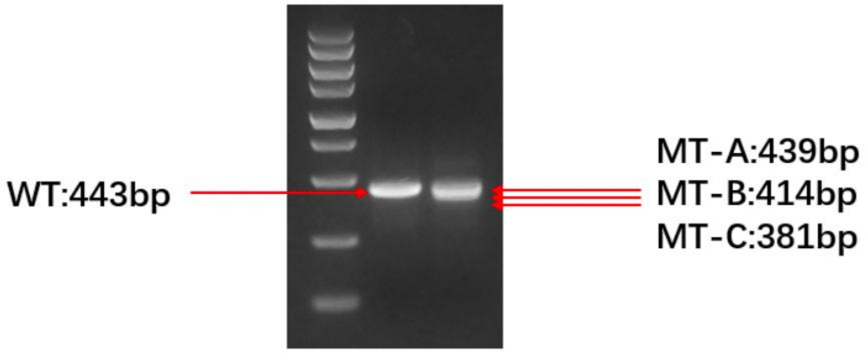

Supplement: Supplementary file 1 [file Presentation_1.PDF]
